# Shannon Entropy and Lévy Statistics for Machine Learning‐Assisted Spectral Sensing in Random Fiber Lasers With Magnetic Core–Shell Nanoscatterers

**DOI:** 10.1002/nap2.70234

**Published:** 2026-08-07

**Authors:** Emanuel P. Santos, Wenyu Du, Edwin D. Coronel, Alyson J. A. Carvalho, G. Palacios, Cecília L. A. V. Campos, Zhijia Hu, Ernesto P. Raposo, Anderson S. L. Gomes

**Affiliations:** ^1^ Departamento de Física Universidade Federal de Pernambuco Recife Pernambuco Brazil; ^2^ Universidade Estadual de Alagoas—Campus VI Maceió Alagoas Brazil; ^3^ State Key Laboratory of Opto‐Electronic Information Acquisition and Protection Technology Information Materials and Intelligent Sensing Laboratory of Anhui Province Key Laboratory of Opto‐Electronic Information Acquisition and Manipulation of Ministry of Education School of Physics and Opto‐electronics Engineering Anhui University Hefei China; ^4^ Centro de Ciências e Tecnologia Universidade Estadual da Paraíba Campina Grande Paraíba Brazil; ^5^ Centro de Ciências Exatas e Sociais Aplicadas Universidade Estadual da Paraíba Patos Paraíba Brazil; ^6^ Laboratório de Física Teórica e Computacional Departamento de Física Universidade Federal de Pernambuco Recife Pernambuco Brazil

**Keywords:** Lévy statistics, neural network, random fiber laser, random laser, Shannon entropy

## Abstract

We investigate the relationship between Shannon entropy and Lévy statistics in a random fiber laser based on a dye‐doped polymer optical fiber with magnetic core–shell ${\text{Fe}}_{3}O_{4}{@\text{SiO}}_{2}$ nanoparticles as scatterers. Spectral measurements acquired below and above the lasing threshold, under varying magnetic fields, are statistically analyzed. Shannon entropy analysis reveals that the intensity fluctuations of each random laser mode, which show the emergence of random laser emission peaks, result in low entropy values. We find a positive correlation between Shannon entropy and the stability index α of the Lévy distribution of output intensities for all magnetic fields and pump energies investigated. As an application, we implement a machine learning approach to classify and predict the magnetic field and pump energy from individual spectra when that information is not available. When the network receives only the spectra as input, the prediction accuracy of the model reaches 69%, but when Shannon entropy is also included as an additional feature, the accuracy increases to 98%. These results demonstrate that Shannon entropy provides relevant complementary information for both the physical characterization of random laser dynamics and the development of efficient real‐time optical sensing systems.

## Graphical abstract

Random laser emission was observed in a polymer optical fiber structure doped with magnetic Fe_3_O_4_@SiO_2_ core–shell nanoparticles. A direct relationship was demonstrated between Shannon entropy and Lévy statistics regarding intensity fluctuations in the emission modes. The analysis indicated a potential application in a proposed magnetic field and energy sensing scheme utilizing machine learning (a neural network).

## 1 Introduction

Shannon entropy is a key concept to measure the information content of a random variable and quantify the average uncertainty in a dataset [1]. Indeed, in Shannon's own words [2], it “plays a central role in information theory as measures of information, choice, and uncertainty”. By considering a discrete random variable X that can take N possible values $\{x_{i}\}$, i=1,...,N, with respective probabilities $\left\{p_{i}=P\left(X=x_{i}\right)\right\}$ subject to the normalization $\sum_{i=1}^{N}p_{i}=1$, Shannon entropy is defined as [1, 2]

(1) $$S=-\sum_{i=1}^{n}p_{i}\;\log\left(p_{i}\right).$$

If the probabilities $p_{i}$ are equally likely, that is, $p_{i}=1/N$ for any i, Shannon entropy is maximum. Conversely, if X assumes values $\left\{x_{i}\right\}$ with heterogeneous probabilities $\{p_{i}\}$, S decreases with respect to the maximum. In the case of a continuous random variable, the sum in Equation (1) is replaced by an integral.

Although Shannon entropy has been defined originally in the context of theory of communication [2], its general formulation makes it currently applied to a wide range of fields, from molecular similarity [3] and financial markets [4] to human communication dynamics [5], biological diversity [6], data analysis, and machine learning [7].

In optics and photonics, Shannon entropy has been explored in a number of systems [8, 9, 10, 11, 12, 13, 14]. In particular, in random lasers (RLs) Shannon entropy has been applied to quantify the degree of randomness in emission sequences, as demonstrated, for example, in dye‐doped nematic liquid crystal films [8]. This approach revealed how the system's unpredictability varies with temperature, enabling the identification of correlations in the temporal series of emission intensities that quantitatively characterize the system's chaotic behavior. On the other hand, in random fiber lasers (RFLs), Shannon entropy has been employed to investigate the presence of spectral correlations, such as in ref. [11], in which a statistical analysis of the emitted spectra is performed to show the existence of correlations and anti‐correlations between spectral peaks, as well as nonlinear mutual information between different regions of the spectra.

RLs and RFLs are optical light sources that can be influenced by external factors, such as temperature [15, 16], magnetic field [17, 18, 19, 20, 21, 22, 23], and electric field [24]. These systems are typically composed of an active gain medium combined with random scatterers, with the latter being responsible for the optical feedback required for laser action [25, 26, 27]. If the medium is embedded with scattering magnetic particles, an external magnetic field influences the RL and RFL emission properties, which might be explored to tune the threshold for random lasing, as theoretically predicted in ref. [17]. In this context, field‐induced photonic Hall effect and photonic magnetoresistance have been observed in a RFL [23], revealing that when a magnetic field acts on the magnetic nanoparticles, both the polarization and scattering cross section of the material are affected, leading to a reduction in the efficiency and an increase in the operational threshold.

Various methods exist to analyze the intensity fluctuations in laser emission for both conventional and random lasers [28, 29, 30, 31, 32, 33, 34]. In RL systems, the discovery [35] that heavy‐tailed Lévy‐like distributions describe well the statistics of emitted intensities around the threshold has opened a new avenue for investigations, which include both experimental and theoretical studies [36, 37, 38, 39, 40, 41, 42, 43, 44, 45]. The probability density function of the family of Lévy α‐stable distributions is given by the inverse Fourier transform of its characteristic function [46], which is expressed as follows:

(2) $$P\left(k\right)=\exp\left\{ik\mu -{\left|ck\right|}^{\alpha }\left[1-i\beta sgn\left(k\right)\Phi \left(k\right)\right]\right\}.$$

The stability index α ∈ (0,2) is the most important parameter since it drives the main statistical properties. Indeed, whereas the borderline value α=2 corresponds to the Gaussian distribution with weak fluctuations, Lévy fluctuation dynamics comprising strong fluctuations are set for 0<α<2. In addition, β ∈ [−1,1] is the asymmetry (skewness) parameter, c>0 is a scale factor, μ is a shift (location) parameter, sgn is the sign function, and $\Phi \left(k\right)=\tan\left[\frac{\alpha \pi }{2}\right]\text{if}\;\alpha \neq 1$, while $\Phi \left(k\right)=-\left(\frac{2}{\pi }\right)\ln\left|k\right|\text{for}\;\alpha =1$. Importantly, the heavy‐tailed asymptotic behavior of the probability distribution of a random variable x described by Lévy statistics has power‐law form, $P\left(x\right)∼1/{\left|x\right|}^{\alpha +1}$, for 0<α<2. In this work, we perform the statistical analysis of the output intensity I at the peak and other wavelengths of the spectral emission, as a function of the applied magnetic field and pump energy. Its probability density P(I) is fitted to the Lévy distribution, showing Gaussian (α=2) or heavy‐tailed (0<α<2) behavior depending on the wavelength, excitation regime, and field. To our knowledge, the study of the influence of the magnetic field on the intensity statistical properties of RLs and RFLs has remained unreported so far.

While the Lévy distribution effectively distinguishes between Gaussian and non‐Gaussian intensity statistics, it does not provide information about the underlying information content of the emission spectra. To address this limitation, we combine the analysis of Lévy statistics with Shannon entropy, enabling a more comprehensive characterization of the emission properties of a random fiber laser with core–shell magnetic nanoparticles. This combined approach reveals complementary aspects of the system, linking the statistical regime of intensity fluctuations to their associated information content, and providing deeper insight into the effects of the applied magnetic field. To further support the experimental findings, numerical simulations based on emission rate equations are also performed. In addition, as an application of this Lévy‐Shannon analysis, we also propose a machine learning approach with the aim of identifying the magnetic field and pump energy of a given spectrum in order to propose a sensing system.

The main novelty of this work lies in the analysis of Shannon entropy, Lévy statistics, and machine learning within a unified framework for the physical characterization of a random laser and for sensing applications. This work demonstrates that Shannon‐Lévy statistical analysis provide correlated and complementary information: the Lévy stability index characterizes the statistical regime of intensity fluctuations, whereas Shannon entropy quantifies the information content and heterogeneity of those fluctuations. The machine learning model presented here works as a detection/sensing strategy in which Shannon entropy is incorporated as an additional feature alongside emission spectra, raising the classification accuracy for magnetic field and pump energy from 69% to 98%. The positive correlation of Shannon‐Lévy statistics establishes a direct link between information theory and the statistical physics of random lasers.

## 2 Experimental Setup

Figure 1 shows the experimental setup for the RFL experiment in an external magnetic field. A pulsed pumping laser (Quantel Q‐SMART450, ∼6ns, 10Hz, beam diameter 6.5μm, pulse‐to‐pulse stability at 532 nm ±4%) operating at 532 nm was used as the excitation energy source, and a half‐wave plate (HWP WPH05M‐532, 532 nm) followed by a polarizing beam splitter (PBS GL10‐A, 350–700 nm) was employed to control the intensity of the incident light. The RFL sample consists of a 3 cm piece of flexible magnetic polymer optical fiber (MPOF), whose cladding material is a copolymer of methyl methacrylate and butyl acrylate with an 80:20 volume ratio, while the core is composed of the same copolymer but with an 85:15 volume ratio. The gain medium (PM597, mass fraction of 0.14%) is uniformly doped into the core with magnetic ${\text{Fe}}_{3}O_{4}{@\text{SiO}}_{2}$ (mass fraction 0.3%) core–shell nanoparticles. The image of the nanoparticle morphology obtained via transmission electron microscopy (TEM) is shown in Supporting Information S1: Figure S1a of the supplementary material (nanoparticle size <100nm). The PM597‐doped MPOF is shown in Supporting Information S1: Figure S1b in a magnified photograph taken with a microscope (Olympus BX51). The sample is placed in a magnet (at the center, to ensure the uniformity of the magnetic field) composed of a pair of coils with power supply (Phywe Stelltrafo mit Gleichrinter) and calibrated using a Phywe digital Teslamenter (sensitivity 10μT, coupled with an axial Hall probe), which generates a homogeneous magnetic field transverse to the fiber. To verify that the field was uniform in the sample region, we probed the entire region using a Hall probe. To eliminate spurious light from the laser source, a long‐pass filter (LP 550) was employed, ensuring that only the RFL emission was collected. For each value of the applied magnetic field (B=0,50,100,200,300mT) and pump energy (E=15,20,25,30,40μJ), 1500 spectra were acquired using a spectrometer (QE65PRO, Ocean Optics, resolution ∼0.4nm, integration time 100ms).

**FIGURE 1:**
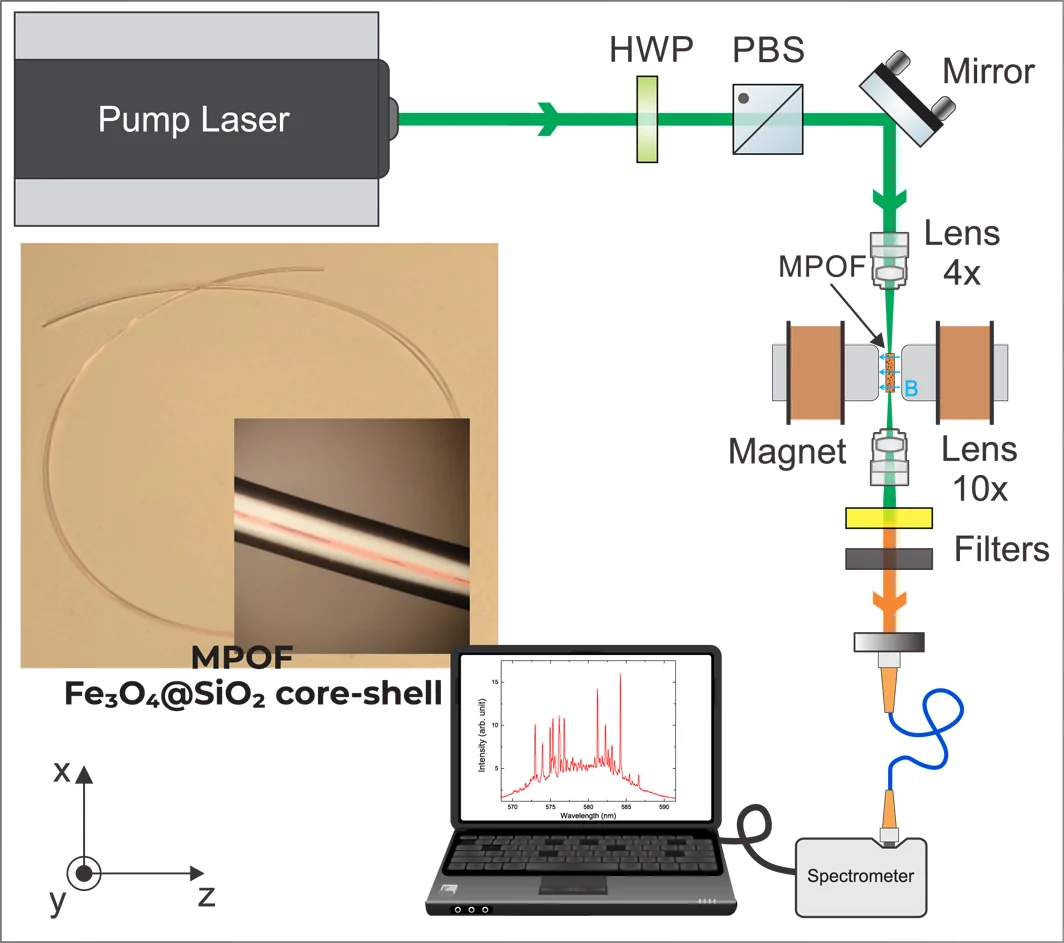
Experimental setup for the statistical study of RFL emission in a magnetic field, showing the pumping laser, half‐wave plate (HWP), polarizing beam splitter (PBS), the magnet responsible for the generation of a homogeneous magnetic field, and the MPOF sample with gain medium doped with magnetic Fe_3_O_4_@SiO_2_ core–shell nanoparticles. The inset depicts a zoom picture of the flexible MPOF.

## 3 Results and Discussion

Figure 2a‐d display some representative spectra and associated Shannon entropy for various values of the applied magnetic field B, in the regimes below and above the random lasing threshold. The spectral range comprises 1448 wavelengths (number of points in the spectrum) within the approximate interval of 567–593 nm. For pump energy E=15μJ in Figure 2a,b, the RFL system operates in the prelasing regime below threshold, for all fields investigated. Indeed, we notice in Figure 2a that the five spectra shown for E=15μJ (one for each value of B) are typically noisy and devoid of lasing modes due to the amplified spontaneous emission (ASE) regime, with absence of pronounced peak intensities. It is also noteworthy that the application of the magnetic field decreases the average emission intensity at each wavelength. In this case, the enhanced polarization of the magnetic nanoparticles for higher B leads to an increased absorption and reduction in the emission, consistent with the photonic magnetoresistance effect [23]. In contrast, for pump energy E=40μJ, Figure 2c shows the presence of several intensity peaks associated with lasing modes, indicating that the random lasing regime has set in above threshold for the fields investigated. The mechanism responsible for the reduced emission in Figure 2a also leads to a decrease in the height of the intensity spikes for higher B.

**FIGURE 2:**
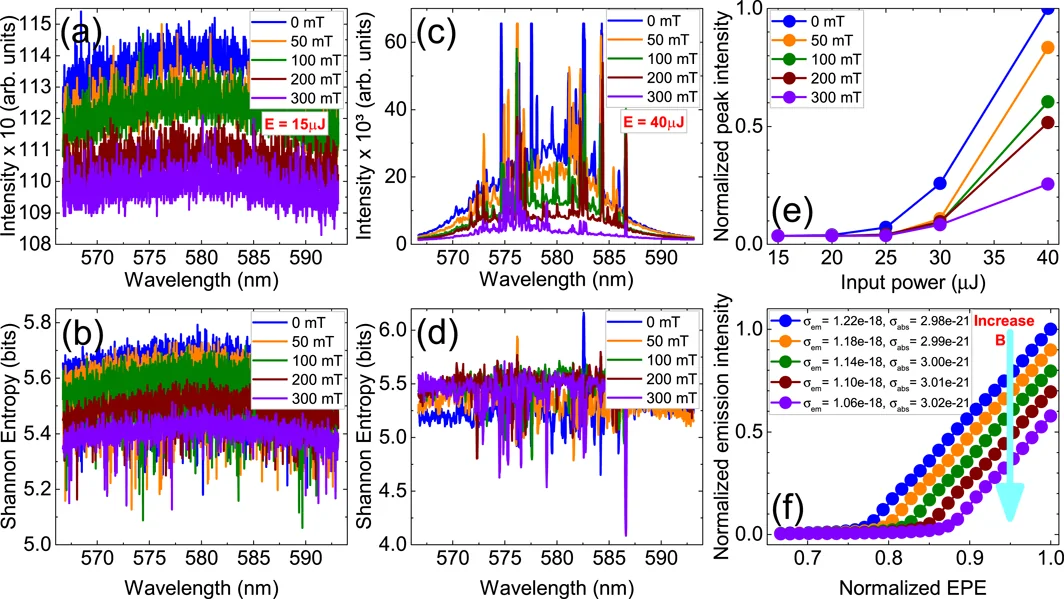
(a–d) Representative spectra (a and c) and Shannon entropy (b and d) as a function of the wavelength for pump energies E=15μJ below threshold (a, b) and E=40μJ above threshold (c, d). Colors indicate the magnetic field values: B=0 (blue), 50mT (orange), 100mT (green), 200mT (red), and 300mT (purple). (e, f) Dependence of the normalized mean peak intensity on the input excitation energy: (e) experimental results for various applied fields; (f) numerical results as a function of the normalized excitation pumping energy (EPE) for several emission ($\sigma_{\text{em}}$) and absorption ($\sigma_{\text{abs}}$) cross‐section values. The blue vertical arrow in (f) indicates the direction of increasing B according to the values of $\sigma_{\text{em}}$ and $\sigma_{\text{abs}}$.

Figure 2b,d present the values of the Shannon entropy S for the same magnetic fields and pump energies considered in Figure 2a,c, respectively. The procedure to calculate S from the experimental datasets was as follows. First, consider a given pair of values of pump energy E and magnetic field B. For each wavelength, 1500 values of intensity (from the 1500 spectra recorded) were classified into small intervals $\left[I_{i},I_{i}+\Delta I\right)$, with $I_{i}$ in the range between the minimum ($I_{\min}$) and the maximum ($I_{\max}$) intensity values and $\Delta I/\left(I_{\max}-I_{\min}\right)=0.01$. Next, from the number of spectra that were set into each interval, we generated a distribution of probabilities, $\{p_{i}\}$, for each wavelength. Lastly, we applied Equation (1) to calculate the Shannon entropy S for each wavelength, with these E and B values (we have used the logarithm to the base 2, so that S is given in bin units or bits). In the sequence, another pair of values of E and B is considered and the procedure is repeated.

For pump energy E=15μJ below threshold, Figure 2b indicates that the Shannon entropy S follows, for each wavelength, an approximate trend of decrease with increasing B. For B=0, the intensity fluctuations over the 1500 spectra are more homogeneous and the probabilities $\{p_{i}\}$ of finding intensity values in the range between $I_{\min}$ and $I_{\max}$ are less dissimilar than in the presence of a field. Indeed, as the field increases from 0 to 300mT, we observe in Figure 2b a small monotonic decrease of S from typically around 5.7 to 5.4 (in bits units). In other words, by applying a magnetic field for fixed pump energy below threshold, not only the emission intensity at each wavelength is reduced, but also the intensity fluctuations get less homogeneous.

A quite different scenario is observed in Figure 2d for pump energy E=40μJ above threshold. First, in contrast to Figure 2b, no clear sign of a monotonic decrease of the Shannon entropy with increasing field is noticed. Rather, one evident feature in Figure 2d is the presence of deep minimum values of S at wavelengths coinciding with the intensity spikes of the random lasing modes in Figure 2c. These entropy minima signalizes that the lasing modes present much stronger and heterogeneous intensity fluctuations, when compared to wavelengths not associated with modes. Interestingly, this meaningful connection between the magnitude and heterogeneity of intensity fluctuations and the values of the applied field and pump energy can also be read off from the statistical analysis of output intensities of the RFL system in terms of Lévy distributions, as we shall see below. We also mention that in Supporting Information S1: Figures S2–S6 (Section S1) of the Supplemental Material, the spectra and Shannon entropy are presented as a function of the magnetic field for the whole set of pump energies investigated (E=15,20,25,30,40μJ).

We now turn to numerically investigate the behavior of the emitted intensity as a function of the magnetic field and pump energy through a model based on rate equations. We respectively denote by n and ρ the excited‐state concentration of particles in the gain medium and the emission energy density of the RFL, which is proportional to the output intensity, yielding the following associated rate equations [47]:

(3) $$\frac{dn}{dt}=\frac{K\left(t\right)N\sigma_{\text{abs}}}{h\nu_{\text{pump}}}-\frac{n}{\tau_{1}}-\beta Nn-\frac{c\sigma_{\text{em}}}{h\nu_{\text{em}}}\;\rho n$$

(4) $$\frac{d\rho }{dt}=-\frac{\rho }{\tau_{2}}+\frac{h\nu_{\text{em}}n}{\tau_{1}}+\gamma_{0}c\sigma_{\text{em}}n\rho .$$

Most parameters values were chosen as in ref. [47]: $N=5\times 10^{21}\;cm^{-3\;}$ is the ground‐state concentration of particles in the gain medium, K(t) is the pump power density at time t, taken as a Gaussian of mean $5\times 10^{-9}\;s$, standard deviation $2.5\times 10^{-18}s$, and amplitude in the range $\left[38,48\right]\times 10^{-2}\;J/cm^{2}$, whose time integral gives the excitation pumping energy (EPE). The terms $h\nu_{\text{pump}}=4\times 10^{19}\;J$ and $h\nu_{\text{em}}=2\times 10^{19}\;J$ denote the photon energies at the pump and emission wavelengths, $\tau_{1}=20\;\mu s$ and $\tau_{2}=10\;\text{ps}$ are the lifetimes of the excited state and photons in the RFL pumped volume. The emission cross‐section adjustment parameter ${0\leq \gamma }_{0}\leq 1$ (for simplicity, $\gamma_{0}=1$ here) accounts for limitations such as competition effects or losses that affect the emission photons leaving the lattice to be counted. When $\gamma_{0}=0$, the nonlinear effect, which is the fundamental signature of the random laser, is lost. The speed of light in the medium is taken as $c=3\times 10^{10}\;\text{cm}/s$, and $\beta N=5\times 10^{3}s^{-1}$ is the rate of cross relaxation.

Moreover, in order to simulate the effect of an applied magnetic field on the RFL, we chose the values of the emission cross section at the wavelength of stimulated emission and absorption cross section at the pump wavelength in the respective ranges $\sigma_{\text{em}}$ ∈ $\left[1.06,1.22\right]\times 10^{-18}{\text{cm}}^{2}$ and $\sigma_{abs}\in \left[2.98,3.02\right]\times 10^{-21}cm^{2}$. We chose a small variation in these parameters because the applied magnetic field intensity is weak (<1T) and the effect is not very strong [23]. Indeed, by writing the absorption coefficient at frequency ω as [24] $\alpha \;\left(\omega \right)=\omega \;\varepsilon_{I}\;\left(\omega \right)\;/\;cn\left(\omega \right)$, where $\varepsilon_{I}$ and n are, respectively, the imaginary part of the complex permittivity and refractive index, we note that an increase in $\varepsilon_{I}$ due to a larger B leads to a larger photon absorption and decrease in the emission, that is, larger $\sigma_{\text{abs}}$ and smaller $\sigma_{\text{em}}$.

Figure 2f shows the output intensity (normalized by its maximum) as a function of the EPE, obtained numerically from Equations (3 and 4). The results for five choices of cross‐section parameters $\left(\sigma_{\text{em}},\sigma_{\text{abs}}\right)$ are displayed, with the blue vertical arrow indicating the increasing magnetic field B. The results corroborate the experimental findings presented in Figure 2e—an analysis of the threshold region can be found in ref. [23], highlighting the dependence of the threshold on the magnetic field. In particular, for higher B, we observe that the onset of the growth of the output intensity also increases, signaling a larger RFL operation threshold, as predicted by Pinheiro in 2008 [17]. Moreover, a field‐induced reduction in the emission is also observed, in agreement with the experimental data.

As discussed in the Introduction section, the family of Lévy α‐stable distributions comprise statistical properties ranging from weakly fluctuating Gaussian behavior, for stability index α=2, to strong fluctuations with asymptotic heavy‐tailed (power‐law) probability density, for 0<α<2. In the following, we analyze the statistical features of output intensities in terms of Lévy distributions as a function of the wavelength, applied magnetic field, and pump energy. We remark that, while the study of the intensity statistics as a function of the pump energy has been well documented (for a review, see, e.g., [25]), to our knowledge the influence of the magnetic field on the intensity statistical properties of RLs and RFLs has remained unreported so far. To compute Lévy statistics, for each set of intensities in each class, we proceed using the quantile‐based McCulloch method [48] as a starting point, with data analysis being refined using maximum likelihood estimation (MLE) [49, 50, 51] to determine the best‐fit parameters of the experimental Lévy distribution.

Figure 3 presents the fits of the experimental data of distribution of peak intensities (blue histograms) to Lévy distributions (red lines), for several magnetic fields (rows) and pump energies (columns). We also tested the fit of these distributions using Gaussian and gamma distributions [43, 52], highlighting the quality of the fit of the Lévy distribution (Supporting Information S1: Table S1). We conclude that using the Lévy fit is more appropriate, based on the metric and the fit presented in Supporting Information S1: Table S1. In each case, the best‐fit values of the stability index α are indicated. We start with the pump energy E=15μJ (first column), for which the RFL system is below threshold for all fields investigated. In this case, we note that the histograms are well described by a single Gaussian for B=300mT (fifth row), and by a statistical mixture of two Gaussians (left and right Gaussians with respective indexes $\alpha_{1}$ and $\alpha_{2}$ equal or near 2) for fields up to 200mT (first to fourth rows). As shown in Figure 2e‐f, the stronger the magnetic field, the higher the energy threshold. Therefore, for B=300mT, the pump energy E=15μJ is much below the threshold than for the other field values. The single Gaussian distribution for the largest field B=300mT in the regime well below threshold arises from spontaneous emission, and is related to weak intensity fluctuations and absence of intensity spikes. On the other hand, as B decreases with fixed E=15μJ, so does the threshold energy. As a consequence, a second maximum in the histograms appears for fields up to B=200mT associated with the ASE mechanism, which becomes relevant as the threshold approaches.

**FIGURE 3:**
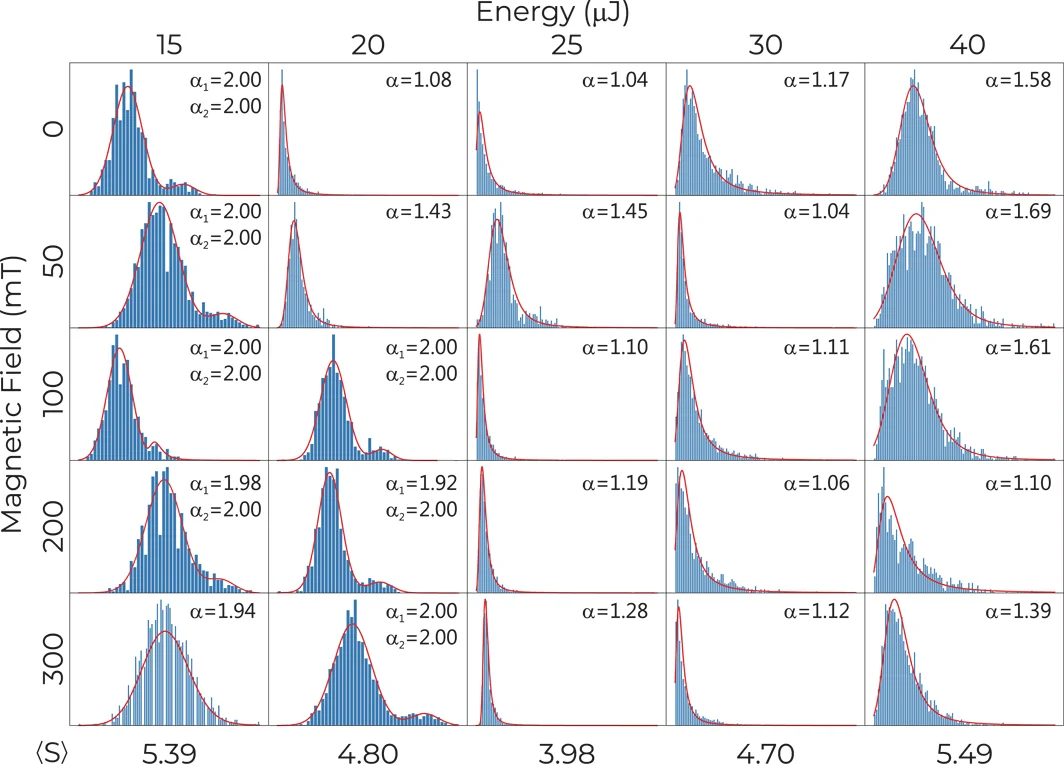
Experimental data of distribution of peak intensities (blue histograms) and fits to Lévy distributions (red lines), for various magnetic fields (rows) and pump energies (columns). Below the threshold, Gaussian distributions with stability index α near or equal to 2 are obtained, manifesting either as a single one well below threshold for the highest field and lowest pump energy, or a statistical mixture of two Gaussians closer to the threshold (left and right ones with respective indexes $\alpha_{1}$ and $\alpha_{2}$). Above the threshold, a Lévy statistical regime sets in with α distinctively smaller than 2. For the highest pump energies, the onset of a trend of increasing α and return to the Gaussian regime takes place. In the bottom line, we display the values 〈S〉 of the Shannon entropy for the peak intensity at each pump energy, averaged over the magnetic fields.

In the second column of Figure 3, the RFL system for E=20μJ is still below threshold for fields B=100,200,300mT (third to fifth rows), and is around the threshold for *B* = 0 and *B* = 50 mT (first and second rows, respectively). For such fields, this fact is reflected in the values of the stability index in the heavy‐tailed Lévy distribution regime (α distinctively smaller than 2). Indeed, low values of α indicate that strong fluctuations and spikes occur in the emitted intensities, related to a heterogeneous distribution of gain among competing modes. On the other hand, in the third column of Figure 3 (E=25μJ), the system is around or above threshold for all fields investigated, and the values of α are consistent with Lévy statistics. Lastly, as the pump energy is enhanced further in the nonlinear regime above threshold for all fields (E=30μJ and E=40μJ, fourth and fifth columns, respectively), although the α values are still relatively low (α≈1.1−1.7), the onset of an upward trend towards the α=2 Gaussian regime can be noticed. The return to the Gaussian emission statistics for very large pump energies has been explained [39, 40, 41] as due to a self‐averaging process in which the gain is so high far above threshold that competition lowers and a more homogeneous distribution of gain among modes takes place, leading to a smoothing of intensity fluctuations and softening of intensity spikes.

The bottom line of Figure 3, just below the histograms, displays the values 〈S〉 of the Shannon entropy for the peak intensity at each pump energy, averaged over the magnetic fields. Interestingly, we note that 〈S〉 follows approximately the same trend of α, that is, 〈S〉 starts with a high value for the lowest pump energy below threshold, in the Gaussian regime with maximum α≈2, consistent with the fact that the noisy spectra are less dissimilar and without spikes. As the pump energy enhances, so does the heterogeneity in the spectra and intensity fluctuations, lowering the values of both 〈S〉 and α. Lastly, the onset of the return to Gaussian statistics for even higher pump energies promotes an increase in the values of both 〈S〉 and α.

The average coefficient of determination is given by $<R^{2}>=<1-\frac{A}{B}>$, where for n values $y_{i}$ (with mean ${\bar{y}}_{i}$) of the distribution and ${\hat{y}}_{i}$ values predicted by the fit, we define $A=\sum_{i=1}^{n}{\left(y_{i}-{\hat{y}}_{i}\right)}^{2}$ and $B=\sum_{i=1}^{n}{\left(y_{i}-{\bar{y}}_{i}\right)}^{2}$, calculated by comparing the Lévy fit with the actual values of the probability distributions, was estimated at $\langle R^{2}\rangle =0.94$. The closer the value is to 1, the better the fit. An alternative method for estimating the uncertainty in the α values was employed by Yadav et al. [53], in which, for each pump fluence, the total set of spectra is divided into smaller, independent subsets, and fits are calculated for both the total set and the subsets to determine the uncertainty represented by an error bar.

Motivated by these findings, we investigate quantitatively the statistical correlation between Shannon entropy and the Lévy stability index. To do this, we calculate the Pearson correlation coefficient, which infers linear correlations between two sets of data, $\left\{x_{i}\right\}$ and $\left\{y_{i}\right\}$. It is defined as follows [54]:

(5) $$C=\frac{\sum \left(x_{i}-\bar{x}\right)\left(y_{i}-\bar{y}\right)}{\sqrt{\sum {\left(x_{i}-\bar{x}\right)}^{2}\sum {\left(y_{i}-{\bar{y}}_{i}\right)}^{2}}}$$

where $\bar{x}$ and $\bar{y}$ denote the mean over $\left\{x_{i}\right\}$ and $\left\{y_{i}\right\}$, respectively. Values C=1 and C=−1 imply, respectively, perfect linear correlation and anticorrelation between the sets, with the degree of linearity varying for intermediate C∈(−1,1). First, we considered $\left\{x_{i}\right\}$ as the set of n=5 values of 〈S〉 shown in the bottom line of Figure 3, and $\left\{y_{i}\right\}$ as the corresponding set of values of α averaged over the fields in column i. In this sense, $\bar{x}$ and $\bar{y}$ represent averages over the pump energies and fields investigated. The result obtained, C=0.75, signals a positive correlation between these sets, in agreement with the discussion above.

Next, we also calculated the Pearson coefficient across wavelengths for specific values of energy pump and magnetic field. This time, the index i in Equation (5) refers to the wavelength points in each spectrum (in total number of n=1448), so that $S_{i}$ and $\alpha_{i}$ are, respectively, the Shannon entropy and stability index for the wavelength i, with given E and B. This calculation involves determining α (from a single Lévy fit) not only for the peak intensity, as in Figure 3, but also for each wavelength. The sets $\{S_{i}\}$ (black lines) and $\left\{\alpha_{i}\right\}$ (red lines) are shown in Figure 4a,b for B=0 and E=25μJ, and for B=200mT and E=25μJ, respectively. We initially note by visual inspection that these sets seem correlated. Indeed, Figure 4c,d plot the sets $\left\{S_{i}\right\}$ and $\left\{\alpha_{i}\right\}$ for the same values of E and B of Figure 4a,b, respectively, showing that a nearly linear relationship exists between them. For both *B* = 0 and E=25μJ, and B=200mT and E=25μJ, we obtained the Pearson coefficient C = 0.80, consistent with a considerable degree of positive linear correlation between S and α.

**FIGURE 4:**
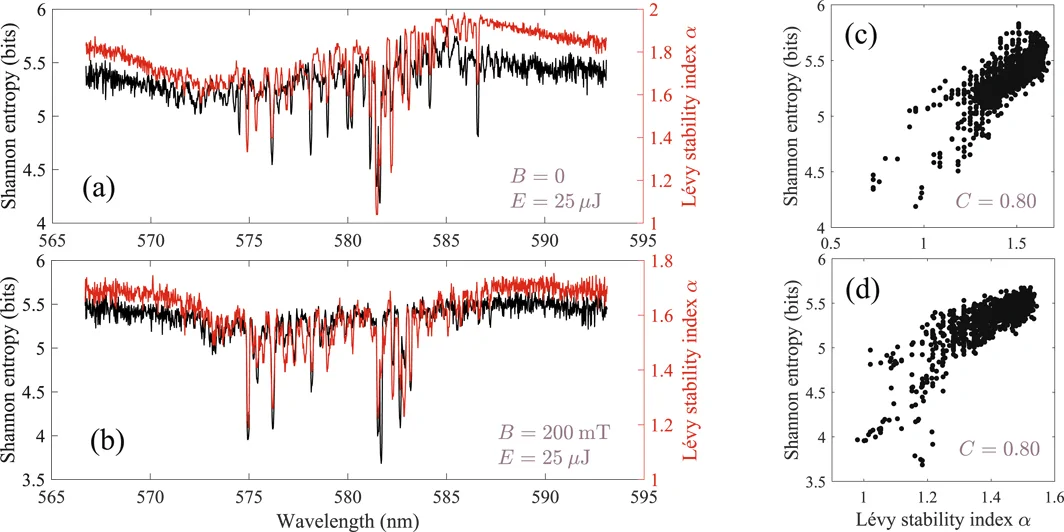
(a) and (b) Shannon entropy (black) and stability index α (red) as a function of the wavelength, for (a) B=0 and E=25μJ, and (b) B=200mT and E=25μJ. (c) and (d) Nearly linear positive correlation between the sets $\left\{S_{i}\right\}$ and $\left\{\alpha_{i}\right\}$, where i labels the wavelengths, for the same energy pump and fields of (a, b), with Pearson coefficient C=0.80 (*p*‐value ∼0, $R^{2}=0.63$, confidence interval of 95% for C: [0.78, 0.81]) in both cases.

Applying a magnetic field to magnetic nanoparticles allows for direct control of the multiple scattering process in the disordered medium of a random laser. Microscopically, the magnetic field polarizes the disordered spins on the surface of the nanoparticles, increasing the effective magnetic moment of the particles, altering the dielectric constant and the absorption coefficient as mentioned earlier. Writing the mean free path of a scattering medium as l=1ρσscat [23, 55], where ρ is the density of particles and $\sigma_{\text{scat}}$ is the scattering cross‐section, then it is necessary that increasing the intensity of magnetic field causes a decrease in the scattering cross‐section. In fact, we have verified this experimentally. Therefore, according to that equation, if $\sigma_{scat}$ decreases, then the mean free path increases, which means that the probability of a photon becoming “lucky”, that is, being amplified in the medium, decreases [38]. Macroscopically, these changes manifest as a reduction in the laser emission intensity, an increase in the lasing threshold, a decrease in intensity fluctuations, and the emergence of photonic magnetoresistance effects, demonstrating that the magnetic field acts as an external parameter capable of modulating both the optical and structural properties of the disordered system.

Shannon entropy quantifies the information content of the probability distribution of intensity fluctuations at each wavelength. In the random laser regime, nonlinear mode competition and amplification via multiple scattering lead to the emergence of rare, strongly amplified spikes, making the intensity distribution increasingly heterogeneous and reducing the entropy of the laser modes. Simultaneously, this gives rise to heavy‐tailed Lévy statistics. These quantities are correlated when spike events occur, as both the Lévy exponent and the Shannon entropy decrease.

## 4 Machine Learning Approach: Spectral Sensing for Input Data Classification

As an application of this synergy between Shannon entropy and Levy statistics, we now introduce a multilayer neural network model to classify the output spectra according to the experimental conditions. Our aim is to devise a machine learning approach that leads to the identification of the magnetic field and pump energy of a given spectrum, even when that information is not available. We first define the “classes” of spectra according to their applied magnetic field B and pump energy E. In this work, because we have considered five values of B (0,50,100,200,and300mT) and five values of E (15, 20, 25, 30, and 40 μJ), 25 classes can be distinguished. For example, Class 1 refers to B=0 and E=15μJ, Class 2 to B=0 and E=20μJ, and so on, up to Class 25 defined for B=300mT and E=40μJ (see Supporting Information S1: Table S2 of the Supporting Information for all classes). Further, as we have recorded 1500 spectra for each class during the experiment, the whole dataset comprises a total of 37500 spectra.

In our *first* approach, we propose to test a multilayer perceptron (MLP) neural network that uses *only* the intensities of the spectra as input information (feature vector) during the learning stage, supervised by the respective classes. The whole set of 1500 spectra for each class are subdivided into 70% that are used in the training procedure, with the remaining ones employed as the validation (15%) and testing (15%) sets to infer the prediction accuracy of the model. Maintaining the same division for the training, validation, and test sets, we implemented a *second* approach that now consists of using the spectral intensity vector (*first* approach) concatenated with the Shannon entropy vector by wavelength as input information for the network. Similarly, in the *third* approach, we concatenate the vector of Levy index by wavelength with the vector of spectral intensities. Finally, the *fourth* approach consists of using all these concatenated vectors (spectrum + Shannon + Levy) as input features for the network. Note that in the *fourth* approach, the feature vector is three times the size of the vector in the *first* approach.

We can compute Shannon entropy S per wavelength by using the whole set of spectra for each class (each E and B), just as described in Figures 2b,d and 4a,b. We term it the total “cumulative” Shannon entropy. In other words, for each class in the total cumulative Shannon entropy, we employ just a single set of spectra containing all spectra recorded. Alternatively, we can also calculate S by considering a number of smaller subsets of spectra for each class, which we call “noncumulative” Shannon entropy. The question that arises is how many spectra per subset we should employ in order to obtain statistically significant results for S. So, to proceed with care, we first considered only a single subset (as in the total cumulative entropy), but varied the number of spectra in this subset to calculate the Shannon entropy. A representative example of this study is shown in Fig. Figure 5a for E=25μJ and B=50mT, in which cumulative S is plotted as a function of the number of spectra used in the single set until the total (other values of E and B lead to similar results). The minimum, maximum, and mean curves in Figure 5a refer, respectively, to the minimum and maximum S for all wavelengths, and the entropy S averaged over the wavelengths. We observe in Figure 5a a rapid initial increase of all (minimum, maximum, and mean) entropies toward saturation as more spectra are considered to obtain S. This indicates that the results converge quite fast to the limit of large number of spectra. We have then considered 5 subsets of 300 spectra each (totaling 1500 spectra) for the calculation of the noncumulative entropy in each class. To avoid data leakage, we also tested other numbers of spectra per subset (10, 50, 150, 250, 275), and the results did not differ significantly. Conversely, Figure 5b shows the minimum, maximum, and mean noncumulative Shannon entropies calculated for each of the 5 subsets of 300 spectra, for the same class of Figure 5a, with E=25μJ and B=50mT. Considering an integration time of 100ms for each spectral acquisition, this is equivalent to having the Shannon entropy updated every 30s in the real‐time experiment dynamics. For a faster sensor, a shorter integration time or fewer spectra for the entropy calculation can be considered. We observe in Figure 5b that, while the minimum entropy presents fluctuations, the maximum and (most importantly) mean S are much more stable over distinct subsets.

**FIGURE 5:**
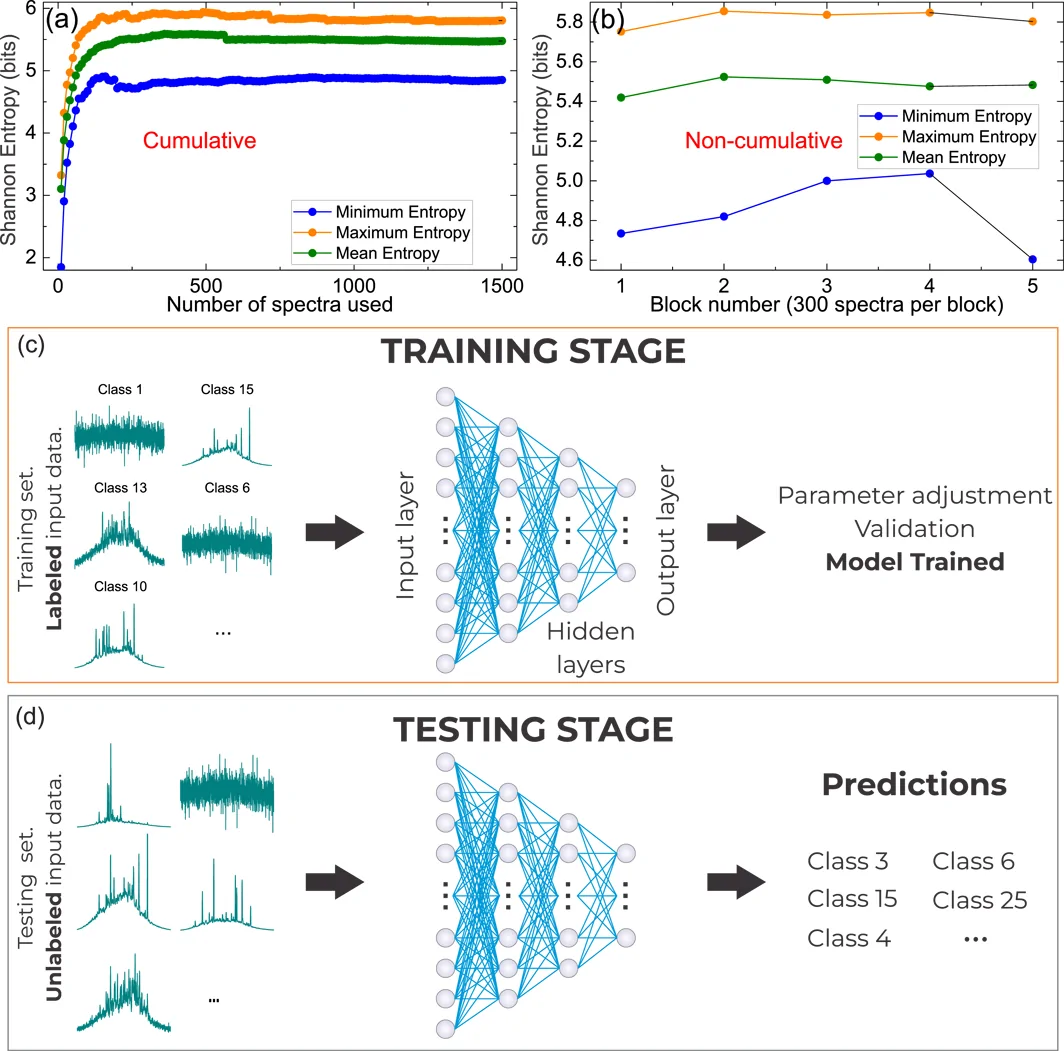
(a) Cumulative Shannon entropy for the class of pump energy E=25μJ and magnetic field B=50mT; (b) noncumulative Shannon entropies as a function, respectively, of the number of spectra used in the calculation and index of subsets containing 300 spectra each, for the class of pump energy E=25μJ and magnetic field B=50mT. Colors depict minimum (blue), maximum (orange), and mean (green) entropies; (c) training stage: supervised learning for classification problem, in which each vector of spectral intensities (*first* approach) or each vector of spectral intensities concatenated with the vector of Shannon entropies by wavelength (*second* approach) or with Levy indices by wavelength (*third* approach), or both concatenated (*fourth* approach), are labeled with their true classes to train the multilayer perceptron with four layers that adjusts the parameters and performs the validation process; (d) during the testing stage, the set reserved for testing enters without labels so that the model makes predictions for each class.

We employed a four‐layer MLP neural network [56], in which the first (input) layer receives the feature vector. The second layer of the MLP (i.e., the first dense layer) has 512 neurons with rectified linear unit (ReLU) activation and dropout of 0.3. The third layer (second dense layer) has 256 neurons with ReLU activation and dropout of 0.3. Finally, the fourth (output) layer has 25 neurons, which is the number of classes in the experiment. This last layer uses the softmax activation function, establishing a probability vector and indicating which class is most likely (see Supplemental Material). We use supervised learning for classification which consists of two basic stages: training and testing. At the training stage (Figure 5c), each input vector (from any of the four approaches) is labeled with its respective class, thus allowing the model to adjust the parameters by comparing the predictions with the real labels, validate with the validation set and generate a trained model with the best parameters (weights and biases), so that during the test stage, Figure 5d, it can make predictions on the spectra not seen during training and which are now unlabeled.

In the *second* approach, we have made the two possibilities for the calculation of the Shannon entropy (cumulative and noncumulative S) available in our experimental acquisition system in order to associate in real time the updated entropy with each new spectra set. In this case, each spectrum within that subset receives the vector of total cumulative or noncumulative entropy values for its subset, which is updated with each new subset (see Section 2 of the Supporting Information S1 for details). In this way, in the *second* approach, each spectrum is assigned not only the intensities but also the entropy as additional information for the input layer. However, for the *third* and *fourth* approaches, we also consider the feature vector of Levy indices by wavelength, thus generating a characteristic vector for each class, which is concatenated to each spectrum within its class. This gives a significant advantage to a classification algorithm, but it may not be useful in the case of real‐time dynamic systems due to the computational complexity of the Lévy distribution, as we will discuss later. However, this is not a problem for the *second* approach, as the Shannon entropy calculation makes sense when done at any time, allowing for a smaller number of spectra, and requiring fewer computational calculations, making it adaptable to real‐time systems.

The ultimate goal of this MLP neural network is to estimate accurately the applied magnetic field and pump energy of any given spectrum when such information is not available. Our results (Figure 6) show that, in the *first* approach using just spectrum intensities as input parameters, a prediction accuracy of 69% to identify the values of the magnetic field and pump energy is obtained for the test set. This indicates that using only spectra in this neural network produces just a reasonably reliable magnetic field and pump energy sensor. However, in the *second* approach, when one considers both the spectrum intensities and associated Shannon entropies (either cumulative or noncumulative) as input parameters, the model yields a mean accuracy of 98% (99% for cumulative and 97% for noncumulative S), indicating an excellent sensing system and classifier. In fact, despite the slightly lower accuracy, the option to test with noncumulative entropy may be safer in order to avoid overfitting and data leakage problems.

**FIGURE 6:**
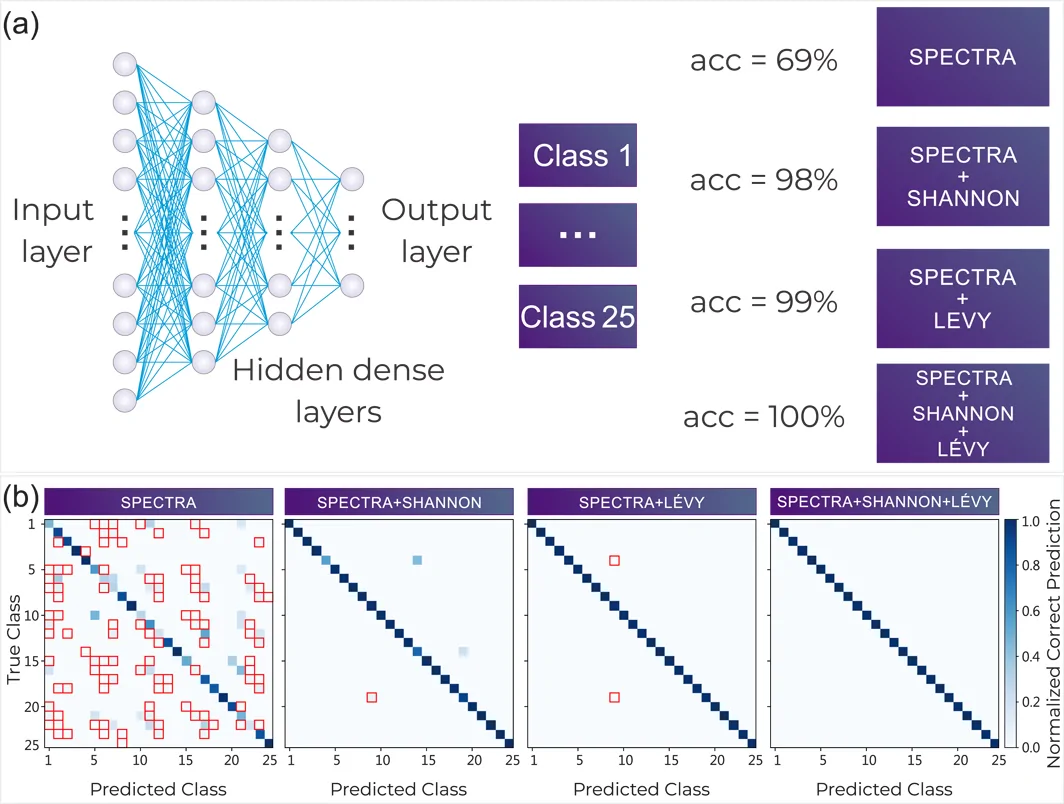
(a) Prediction accuracy (acc) of the test set is 69% when using only spectrum intensities as input parameters (*first* approach), 98% on average with both spectra and Shannon entropy as input (99% for cumulative and 97% for noncumulative S; *second* approach), 99% for the *third* approach, and 100% in *fourth* approach. (b) The confusion matrices reveal the correct (normalized) predictions in blue scale. We highlight, using red squares, the classes with practically negligible errors on the color scale, which are less than 5%. We also tested using only 10 spectra per subset (noncumulative entropy, sensor updated every 1s using 100ms time integration), which resulted in 96% accuracy.

As we mentioned before, approaches that use Lévy indices require greater care when proposing real‐time applications. The *third* approach shows 99% accuracy and *fourth* approach shows 100% accuracy, which was expected since the Levy index vectors are very distinct between classes. Initially, we could propose a classification system based solely on Levy index; however, this is not feasible for real‐time or fast dynamic applications for the following reason. Levy statistics require a considerable number of spectra to reliably analyze and distinguish the intensity fluctuations between classes, and requires greater computing power due to the search for the best fit in each case to obtain the α indices at all wavelengths. This contrasts with Shannon entropy calculation, which is computationally lightweight and can be performed reliably with a small number of spectra, can be updated in real time within seconds or less, depending on the spectral acquisition time. Time measurements between the two approaches (Shannon and Lévy) can be viewed in Supporting Information S1: Table S3 of the supplementary material, in which we show that the time to calculate Shannon entropy ($t_{s}$) is much less than the time to calculate Lévy index ($t_{\alpha }$), with $t_{\alpha }/t_{s}\;∼\;10^{6}$. Therefore, we consider the *second* approach to be the most promising for practical dynamic sensing systems, since the accuracy is above 97%.

Our results show that the improvement associated with including the Shannon entropy (Supporting Information S1: Figure S7) and Levy statistics as an additional feature in the model can be relevant for enhancing the accuracy in the identification of the applied magnetic field and pump energy of spectra. This feature makes the present MLP network a promising tool for use as a sensing system in dynamic time during experimental measurements. We also considered classic machine learning models for the same analysis (Section 3 and Supporting Information S1: Table S4 of the supplementary material). The results were very close, which leads to the possibility of using either of them for our data. We opted to represent these final results found by MLP due to its ability to universalize to other systems, as well as greater power in algorithm architecture by the user, so that the number of layers, neurons, and other hyperparameters are adjustable and can be adapted to various different systems.

## 5 Conclusion

In this work, we have investigated the effects of an applied magnetic field and pump energy on the Shannon entropy and emission statistics of a random fiber laser doped with magnetic core–shell nanoparticles. While the study of Lévy intensity statistics as a function of the excitation energy has been reported in the literature, the combined influence with the application of an external magnetic field, added to the combination with Shannon entropy, provided novel understanding in the field of random fiber lasers. We found a positive correlation between Shannon entropy and the Lévy stability index α, which opens new perspectives of study within theoretical statistical physics and experimental physics by combining information theory, statistics of complex systems, and photonics of disordered media. Furthermore, we introduced a multilayer neural network model to classify the emission spectra according to the experimental conditions. We have found that when Shannon entropy is included as an additional feature (*second* approach in the text), the prediction accuracy of the model can increase up to 98%. This result opens an interesting possibility of using the proposed network as a real‐time sensing system. In addition to establishing a correlation, the combined Shannon and Lévy entropy analysis reveals a direct link between the information content of spectral fluctuations and the underlying statistical regime of emissions, providing a physical signature of the transition between Gaussian and non‐Gaussian dynamics, defining each class in a virtually unmistakable way, that is, each experimental condition is very well detected by the trained neural network.

This methodology can also be potentially applied to train a complex neural network to infer the parameters of the Lévy distribution of emitted intensities by taking Shannon entropy as input information. Moreover, since Shannon entropy is important for communications and cryptography [2, 57, 58], and applications in these fields have already been proposed using lasers and random lasers [59, 60, 61], then analyzing Shannon entropy of each mode can be relevant. For example, by selecting one of the modes as a message transmitter or decoder, the message can become more or less secure, or contain more or less information, depending on the value of the corresponding Shannon entropy.

Furthermore, the results presented here can serve as a basis for future work involving other samples, experimental configurations, and distinct initial conditions. Each parameter can be varied to determine the optimal conditions for developing faster and more precise sensors.

## Author Contributions

**Emanuel P. Santos:** conceptualization, investigation, methodology, writing – original draft, writing – review and editing, formal analysis. **Wenyu Du:** investigation, writing – original draft, writing – review and editing, methodology, formal analysis. **Edwin D. Coronel:** investigation, writing – original draft, writing – review and editing, formal analysis, methodology. **Alyson J. A. Carvalho:** investigation, writing – original draft, writing – review and editing, methodology, formal analysis. **G. Palacios:** investigation, writing – original draft, writing – review and editing, methodology, formal analysis. **Cecília L. A. V. Campos:** investigation, writing – original draft, writing – review and editing, methodology, formal analysis. **Zhijia Hu:** investigation, writing – original draft, writing – review and editing, methodology, formal analysis. **Ernesto P. Raposo:** writing – original draft, investigation, writing – review and editing, methodology, formal analysis. **Anderson S. L. Gomes:** writing – original draft, investigation, writing – review and editing, methodology, formal analysis.

## Supporting information


Supporting Information S1

## Data Availability

The data that support the findings of this study are available from the corresponding author upon reasonable request.
