# Supplementary material for: Shannon Entropy and Lévy Statistics for Machine Learning‐Assisted Spectral Sensing in Random Fiber Lasers With Magnetic Core–Shell Nanoscatterers
Source: Nanophotonics. 2026 Aug 7;15(15):e70234. doi: 10.1002/nap2.70234 (PMC13642104; doi:10.1002/nap2.70234)
Supplement: Supplementary file 1 [file NAP2-15-e70234-s001.docx]

**Shannon Entropy and Lévy Statistics for Machine Learning-Assisted Spectral Sensing in Random Fiber Lasers With Magnetic Core-Shell Nanoscatterers**

Emanuel P. Santos^1,2*^, Wenyu Du^3^, Edwin D. Coronel^1^, Alyson J. A. Carvalho^1,4^, G. Palacios^1,5^, Cecília L. A. V. Campos¹, Zhijia Hu³^,†^, Ernesto P. Raposo^6,‡^, Anderson S. L. Gomes^1,§^

^1^Departamento de Física, Universidade Federal de Pernambuco, 50670-901, Recife, PE, Brazil.

²Universidade Estadual de Alagoas, 57025-602, Maceió, AL, Brazil.

³State Key Laboratory of Opto-Electronic Information Acquisition and Protection Technology, Information Materials and Intelligent Sensing Laboratory of Anhui Province, Key Laboratory of Opto-Electronic Information Acquisition and Manipulation of Ministry of Education, School of Physics and Opto-electronics Engineering, Anhui University, Hefei, 230601, China.

^4^Centro de Ciências e Tecnologia, Universidade Estadual da Paraíba, 58429-500, Campina Grande, PB, Brazil.

^5^Centro de Ciências Exatas e Sociais Aplicadas, Universidade Estadual da Paraíba, 58706-550, Patos, PB, Brazil.

^6^Laboratório de Física Teórica e Computacional, Departamento de Física, Universidade Federal de Pernambuco, 50670-901 Recife, PE, Brazil.

*emanuel.pinheiro@ufpe.br

†zhijiahu@ahu.edu.cn

‡ernesto.raposo@ufpe.br

§anderson.lgomes@ufpe.br

1. **Nanoparticles (NPs), Magnetic Polymer Optical Fiber (MPOF), Spectra, Shannon entropy for various magnetic fields and pump energies and goodness-of-fit for Lévy distribution**

Figure S1. (a) TEM image of $Fe_{3}O_{4}@SiO_{2}$ NPs (black scale bar, 100 nm), inset: dynamic variation of the $Fe_{3}O_{4}@SiO_{2}$ NPs under magnetic field – reproduced from [1] with permission under CCBY 4.0 license. (b) Photograph of MPOF PM597-doped, inset: zoom from microscope Olympus BX51.

In Figs. S2-S6 below, we present some spectra [(a)] and corresponding Shannon entropy [(b)] as a function of the wavelength, for all applied magnetic fields (*B* = 0, 50, 100, 200, 300 mT) and pump energies (*E* = 15, 20, 25, 30, 40 *μ*J) investigated.

Figure S2. Representative spectra (left) and Shannon entropy (right) as a function of the wavelength for pump energy $E = 15 \mu J$. Colors in the same order that the Figure 2 of main article.

Figure S3. Representative spectra (left) and Shannon entropy (right) as a function of the wavelength for pump energy $E = 20 \mu J$. Colors in the same order that the Figure 2 of main article.

Figure S4. Representative spectra (left) and Shannon entropy (right) as a function of the wavelength for pump energy $E = 25 \mu J$. Colors in the same order that the Figure 2 of main article.

Figure S5. Representative spectra (left) and Shannon entropy (right) as a function of the wavelength for pump energy $E = 30 \mu J$. Colors in the same order that the Figure 2 of main article.

Figure S6. Representative spectra (left) and Shannon entropy (right) as a function of the wavelength for pump energy $E = 40 \mu J$. Colors in the same order that the Figure 2 of main article.

Table S1. Comparison among Lévy (red fit), Gamma (blue fit) and Gaussian fits (green fit).

2. **Details of the neural network model and data analysis**

The 25 classes of spectra mentioned in the main text are shown in Table S2 below. Each class is defined by the values of the applied magnetic field *B* and pump energy *E*.

Table S2. Definition of the classes of spectra.

|  | 15 *μ*J | 20 *μ*J | 25 *μ*J | 30 *μ*J | 40 *μ*J |
| --- | --- | --- | --- | --- | --- |
| 0 | Class 1 | Class 2 | Class 3 | Class 4 | Class 5 |
| 50 mT | Class 6 | Class 7 | Class 8 | Class 9 | Class 10 |
| 100 mT | Class 11 | Class 12 | Class 13 | Class 14 | Class 15 |
| 200 mT | Class 16 | Class 17 | Class 18 | Class 19 | Class 20 |
| 300 mT | Class 21 | Class 22 | Class 23 | Class 24 | Class 25 |

The dataset used in the present study consists in emission spectra obtained from 25 independent experimental conditions, each one associated with a distinct class given in Table S1. Each class contains 1500 individual spectra (samples), with 1448 intensity points (features) each, corresponding to the wavelengths.

To characterize not only the spectral intensity profiles but also their statistical fluctuations, Shannon entropy was computed independently for groups of spectra. In particular, for the non-cumulative entropy the whole dataset of 1500 spectra in each class was divided into 5 subsets of 300 spectra (is also possible to use more subsets with fewer spectra each). For each subset, the entropy was calculated separately at each wavelength, ensuring that the entropy estimation is local and non-cumulative (and avoiding data leakage). This procedure results, for each subset, in an entropy vector of length 1448, corresponding to the non-cumulative entropy at each wavelength. The entropy vector of a subset is then assigned to all spectra belonging to that subset. Two feature representations were constructed: in the first case (first approach), only the raw spectral intensities were used, leading to a feature vector of dimension 1448 per spectrum. In the second case (second approach), the raw spectrum was concatenated with its corresponding entropy vector, resulting in an augmented feature vector of dimension 2896 that jointly encodes spectral and statistical information.

The third approach described in the article was implemented considering all 1500 spectra for each class, and the Lévy index is calculated at each wavelength (Levy index feature vector). That is, since we have 1448 wavelengths, the vector of Lévy indices will have 1448 points, as shown in Figures 4(a) and 4(b). Then, the Levy index feature vector is concatenated with the feature vector of intensities. Thus, the fourth approach, concatenating the vectors of spectral intensities, Shannon entropy, and Lévy indices will result in three times the number of features in the input layer (1448 + 1448 + 1448 = 4344) compared to the first approach. The rest of the model remains the same, maintaining the hyperparameters and subsequent layers with the same sizes. In these last cases, to train the network, it is necessary to accumulate the 1500 spectra, take the vector of Lévy indices (which can be computationally heavy and inefficient for fast-time dynamics – Table S3), and then concatenate it to the spectra feature vector.

Table S3. Here we consider all spectral intensities of each class for the 575 nm mode to calculate Shannon entropy and the Lévy $\alpha$ index. The methodology used was to use Python through the Jupyter Project using the scipy.stats library (“from scipy.stats import levy_stable, entropy”). Because the entropy calculation is much simpler than the Lévy calculation, the time to calculate each differs significantly – compare the last two columns.

| **Class** | **Computer configuration (CPU, RAM, GPU)** | **Shannon Entropy S at 575 nm (bits)** | $\boldsymbol{\alpha}$ **index (575 nm)** | **Time to compute** $\boldsymbol{S}_{\boldsymbol{575 nm}}$ **(s)** | **Time to compute** $\boldsymbol{\alpha}_{\boldsymbol{575 nm}}$ **(s)** |
| --- | --- | --- | --- | --- | --- |
|  | | | | | |
| 1 | Intel Core i7 10750H 2.60 GHz, 16.0 GB, 6 GB | 5.577785 | 1.642250 | 0.000640 | 468.437648 |
| 2 | Intel Core i7 10750H 2.60 GHz, 16.0 GB, 6 GB | 4.875969 | 1.046434 | 0.000692 | 916.810078 |
| 3 | Intel Core i7 10750H 2.60 GHz, 16.0 GB, 6 GB | 5.321147 | 1.379284 | 0.000630 | 1131.446447 |
| 4 | Intel Core i7 10750H 2.60 GHz, 16.0 GB, 6 GB | 5.215627 | 1.626514 | 0.000638 | 1040.185404 |
| 5 | Intel Core i7 10750H 2.60 GHz, 16.0 GB, 6 GB | 5.536517 | 1.514797 | 0.000734 | 803.889702 |
| 6 | Intel Core i7 10750H 2.60 GHz, 16.0 GB, 6 GB | 5.456669 | 1.651543 | 0.000704 | 419.678090 |
| 7 | Intel Core i7 10750H 2.60 GHz, 16.0 GB, 6 GB | 5.187325 | 1.485573 | 0.000641 | 583.191253 |
| 8 | Intel Core i7 10750H 2.60 GHz, 16.0 GB, 6 GB | 5.430436 | 1.485670 | 0.000588 | 506.377750 |
| 9 | Intel Core i7 10750H 2.60 GHz, 16.0 GB, 6 GB | 5.473381 | 1.544268 | 0.000603 | 954.429808 |
| 10 | Intel Core i7 10750H 2.60 GHz, 16.0 GB, 6 GB | 5.258192 | 1.389873 | 0.000607 | 971.501863 |
| 11 | Intel Core i7 10750H 2.60 GHz, 16.0 GB, 6 GB | 5.537990 | 1.688620 | 0.000604 | 932.560404 |
| 12 | Intel Core i7 10750H 2.60 GHz, 16.0 GB, 6 GB | 5.167840 | 1.565322 | 0.000597 | 432.480424 |
| 13 | Intel Core i7 10750H 2.60 GHz, 16.0 GB, 6 GB | 5.554606 | 1.403309 | 0.000600 | 883.757239 |
| 14 | Intel Core i7 10750H 2.60 GHz, 16.0 GB, 6 GB | 5.081207 | 1.106173 | 0.000604 | 2139.576853 |
| 15 | Intel Core i7 10750H 2.60 GHz, 16.0 GB, 6 GB | 5.184906 | 1.424949 | 0.000603 | 1021.744909 |
| 16 | Intel Core i7 10750H 2.60 GHz, 16.0 GB, 6 GB | 5.523344 | 1.813508 | 0.000601 | 632.024683 |
| 17 | Intel Core i7 10750H 2.60 GHz, 16.0 GB, 6 GB | 5.544451 | 1.618914 | 0.000597 | 517.331522 |
| 18 | Intel Core i7 10750H 2.60 GHz, 16.0 GB, 6 GB | 4.074720 | 1.067203 | 0.000601 | 1694.214470 |
| 19 | Intel Core i7 10750H 2.60 GHz, 16.0 GB, 6 GB | 4.992429 | 1.298592 | 0.000611 | 721.046727 |
| 20 | Intel Core i7 10750H 2.60 GHz, 16.0 GB, 6 GB | 5.539955 | 1.488232 | 0.000585 | 1318.648402 |
| 21 | Intel Core i7 10750H 2.60 GHz, 16.0 GB, 6 GB | 5.399628 | 1.931660 | 0.000597 | 707.309331 |
| 22 | Intel Core i7 10750H 2.60 GHz, 16.0 GB, 6 GB | 5.456647 | 1.550501 | 0.000599 | 424.544885 |
| 23 | Intel Core i7 10750H 2.60 GHz, 16.0 GB, 6 GB | 4.709408 | 1.427763 | 0.000617 | 466.301513 |
| 24 | Intel Core i7 10750H 2.60 GHz, 16.0 GB, 6 GB | 5.684728 | 1.470920 | 0.000591 | 721.341099 |
| 25 | Intel Core i7 10750H 2.60 GHz, 16.0 GB, 6 GB | 5.391125 | 1.391834 | 0.000605 | 703.359677 |
|  | | | | | |
| 1 | AMD Ryzen 5 5500U 2.10 GHz, 16.0 GB, 496 MB | 5.5778 | 1.6422 | 0.000753 | 695.343276 |
| 2 | AMD Ryzen 5 5500U 2.10 GHz, 16.0 GB, 496 MB | 4.8760 | 1.0464 | 0.000938 | 1343.611061 |
| 5 | AMD Ryzen 5 5500U 2.10 GHz, 16.0 GB, 496 MB | 5.5365 | 1.5148 | 0.000802 | 1287.130661 |
| 8 | AMD Ryzen 5 5500U 2.10 GHz, 16.0 GB, 496 MB | 5.4304 | 1.4857 | 0.000672 | 744.644212 |
| 9 | AMD Ryzen 5 5500U 2.10 GHz, 16.0 GB, 496 MB | 5.4734 | 1.5443 | 0.000662 | 1398.126952 |
| 12 | AMD Ryzen 5 5500U 2.10 GHz, 16.0 GB, 496 MB | 5.1678 | 1.5653 | 0.000660 | 627.936826 |
| 15 | AMD Ryzen 5 5500U 2.10 GHz, 16.0 GB, 496 MB | 5.1849 | 1.4249 | 0.000782 | 1515.262398 |
| 18 | AMD Ryzen 5 5500U 2.10 GHz, 16.0 GB, 496 MB | 4.0747 | 1.0672 | 0.000719 | 2609.734593 |
| 24 | AMD Ryzen 5 5500U 2.10 GHz, 16.0 GB, 496 MB | 5.6847 | 1.4709 | 0.000793 | 1098.263082 |

Each of the 25 class has 1500 spectra, and so the complete dataset comprises 37500 spectra. The total dataset was then split into training, validation, and test sets using stratified sampling to preserve class balance. 70% of the data (26250 spectra) were used for training, while the remaining 30% were equally divided into validation and test sets, each containing 5625 spectra. To evaluate the impact of entropy-based features on classification performance, a multilayer perceptron (MLP) neural network was employed.

The neural network consists of an input layer whose size matches the dimensionality of the feature vector, followed by two fully connected hidden layers with 512 and 256 neurons, respectively. Both hidden layers use the ReLU activation function and are followed by dropout layers, with a dropout rate of 0.3 to reduce overfitting. The output layer contains 25 neurons, corresponding to the number of classes, and uses the softmax activation function. The network was trained using the Adam optimizer and categorical cross-entropy as the loss function. Class labels were encoded using a one-hot representation. Training was performed for up to 50 epochs with a batch size of 128, and early stopping was applied with a patience of 10 epochs, monitoring the validation loss and restoring the model weights corresponding to the best validation performance. The models were trained and evaluated under the same network. During the training and validation phase, we monitored accuracy and loss over the epochs, as shown in Fig. S6.

Fig. S7. Training versus validation accuracy and loss.

The accuracy is calculated as the proportion of correctly predicted samples on the set, i.e., accuracy = (number of correct predictions)/(number of total predictions). The loss quantifies how wrong the predictions are based on the loss function of the model, which in our case corresponds to the categorical cross-entropy for classification problem. The trained models were evaluated on the independent test set to assess their generalization performance.

Algorithm summary:

1. Dataset organization and class definition (25 classes × 1,500 spectra each = 37,500 spectra).
2. Selection of one of the four approaches (spectra, spectra+Shannon, spectra+Lévy, spectra+Shannon+Lévy) and construction of the feature vector for each approach according to the procedure described above.
3. Data splitting into training (70%), validation (15%), and testing (15%) sets.
4. Neural network construction (the model used is described above and in the article).

5.	Training and validation (hyperparameters are described above).

6.	Final evaluation on the test set by calculating accuracy (described above).

3. **Results from other models**

Table S4. Comparison among models.

| Model | Parameters | Result |
| --- | --- | --- |
| MLP (current) | Described in the paper | **Spectra = 0.69**  **Spectra+Shannon= 0.98** |
| Random Forest | n_estimators = 200 | **Spectra = 0.67**  **Spectra+Shannon = 0.97** |
| Support Vector Machine with RBF Kernel | kernel='rbf'  C=10 | **Spectra = 0.66**  **Spectra+Shannon = 0.96** |
| K - Nearest neighbors | n_neighbors = 5 | **Spectra = 0.58**  **Spectra+Shannon = 0.96** |

Random Forest is one of the best-known and classic machine learning algorithms  [2]. Although based on decision trees, it is not strictly a single tree, but rather an ensemble of multiple decision trees. Each tree is constructed from random samples of the data (with replacement) and a random subset of features. At each node of the tree, a decision is made based on a feature, dividing the data into subsets. The algorithm combines the results of all the trees usually by voting to produce a final prediction that is more robust and has less overfitting than a single tree. The number of trees in the forest is a hyperparameter, usually called n_estimators in implementations (e.g., scikit-learn). In our case, we choose n_estimators = 200.

Random Forest library to python:

(https://scikit-learn.org/stable/modules/generated/sklearn.ensemble.RandomForestClassifier.html)

The Support Vector Machine (SVM) is another classic supervised learning algorithm. It works by finding a decision boundary (hyperplane) that best separates different classes while maximizing the distance from the hyperplane to the closest points from each class (the support vectors). For data that is not linearly separable, SVM uses kernel functions to transform the data into a higher-dimensional space where separation becomes possible. The radial basis function (RBF) kernel is one of the most popular kernels; it measures similarity between data points based on their distance, allowing SVM to capture complex, non-linear patterns. The parameter C controls the trade-off between having a smooth decision boundary and correctly classifying training points — a high C (like C=10) prioritizes correct classification of all training examples, which can lead to a more complex boundary.

SVM library to python:

https://scikit-learn.org/stable/modules/svm.html

K-Nearest Neighbors (KNN) is an intuitive and simple machine learning algorithm that can be used for classification tasks. The KNN model stores all training data and makes predictions at query time based on similarity. For classification, the algorithm identifies the “K” closest data points (neighbors) to the new sample using a distance metric (typically Euclidean distance) and assigns the majority class among those neighbors. The hyperparameter K (number of neighbors, or, “n_neighbors”) controls the bias-variance trade-off. In our case, we used n_neighbors = 5, which is a common default that balances local sensitivity and robustness to outliers.

KNN library to python:

https://scikit-learn.org/stable/modules/generated/sklearn.neighbors.KNeighborsClassifier.html

REFERENCES

[1]	W. Du et al., Observation of the photonic Hall effect and photonic magnetoresistance in random lasers, Nat Commun **15**, 1 (2024).

[2]	A. Géron, *Hands-on Machine Learning with Scikit-Learn, Keras, and TensorFlow* ( O’Reilly Media, Inc., 2022).
